# Addressing antimicrobial resistance by improving access and quality of care—A review of the literature from East Africa

**DOI:** 10.1371/journal.pntd.0009529

**Published:** 2021-07-22

**Authors:** Kathrin Loosli, Alicia Davis, Adrian Muwonge, Tiziana Lembo

**Affiliations:** 1 The Boyd Orr Centre for Population and Ecosystem Health, Institute of Biodiversity, Animal Health & Comparative Medicine, College of Medical, Veterinary & Life Sciences, University of Glasgow, Glasgow, United Kingdom; 2 School of Social and Political Sciences/Institute of Health and Wellbeing, University of Glasgow, Glasgow, United Kingdom; 3 The Roslin Institute, Royal (Dick) School of Veterinary Studies, University of Edinburgh, Edinburgh, United Kingdom; JH-Institute of Molecular Medicine, INDIA

## Abstract

Universal access to healthcare, including quality medicines, is a fundamental human right but is still out of reach for many in low- and middle-income countries (LMICs). An existing framework capturing variability of access to healthcare in low-resource settings includes the 5 dimensions: availability, accessibility, affordability, adequacy, and acceptability. This framework encompasses key components, including health infrastructure and means to access it as well as service organisation, costs, and factors that influence users’ satisfaction. However, in reality, the effectiveness of accessed healthcare is measured by the likelihood of a positive outcome. We therefore propose an expansion of this framework to include an additional dimension, “aspects of quality,” incorporating quality, which critically influences the ability of the accessed services to generate optimal health outcomes. Within this framework, we explore literature from East Africa likely relevant to a range of LMIC contexts, mainly focusing on the provision of widely used antimicrobials such as antimalarials and antibiotics. We argue that major inadequacies exist across all 6 dimensions of access and quality of drugs and their provision. While the global focus is on curbing excessive antimicrobial use to tackle the antimicrobial resistance (AMR) crisis, major constraints around access shape patients’ health-seeking decisions leading to potentially problematic practices that might exacerbate the AMR problem. We advocate for a holistic approach to tackling these inadequacies, encompassing all dimensions of access and quality of healthcare in order to improve health outcomes while simultaneously counteracting the AMR crisis.

## Introduction

### Background

In the last decade, sub-Saharan Africa has consistently registered a high burden of disease in both humans [1] and livestock [2], which is consistent with patterns observed in low- and middle-income countries (LMICs) more broadly. Some of the most prevalent and devastating illnesses are of infectious nature [1,2]. Noticeably, infections play a major role in maternal and child mortality, being the cause of 11% of maternal and 25% of newborn deaths, respectively [3]. Surgical site infections (SSIs) are a substantial cause of deaths in hospitals [4]. Cesarean sections constitute one of the most performed surgeries in sub-Saharan Africa [5,6], and SSIs represent the main complications of this procedure. This disease burden has direct effects on economic growth and constrains the attainment of key Sustainable Development Goals (SDGs) [7]. Beyond the mortality burden, disease morbidity has a high socioeconomic impact on individuals and communities [7]. Indeed, it is often remote or poor communities who are the most disadvantaged by health disparities and inequalities [8].

Antimicrobials save thousands of lives every year and reduce disease mortality and morbidity all over the world. Together with biosecurity, antimicrobials have also transformed livestock production and enhanced food security, especially in parts of the world where human health and well-being are strongly dependent on livestock [9]. Yet, the antimicrobial resistance (AMR) crisis has switched the focus to excessive antimicrobial use, creating tension between the need to improve universal access to essential drugs and the global push to reduce their use [10].

### Frameworks for examining access to quality healthcare

Penchansky and Thomas (1981) introduced 5 dimensions of access [11], which Obrist and colleagues (2007) modified and adapted as an analytical framework “to explore and improve access to health care in resource-poor countries, especially in Africa” [12]. These 5 dimensions are availability, accessibility, affordability, adequacy, and acceptability, and encompass a wide range of factors critical to optimal health care, from the existence and accessibility of health infrastructure in a broad sense (e.g., health centres, pharmacies, personnel, diagnostics, and drugs) to their organisation, acceptability to patients, and associated costs.

Access alone, however, does not guarantee positive health outcomes. To achieve this goal, the accessed care must be of good quality, i.e., possess the ability to treat and cure patients. Quality of care is defined by the World Health Organization (WHO) as “the degree to which health services for individuals and populations increase the likelihood of desired health outcomes and are consistent with current professional knowledge” [13]. In their report “Delivering Quality Health Services,” they emphasise the need for “providing effective, safe, people-centred care that is timely, equitable, integrated and efficient” [13]. All these aspects are influenced by the quality of medicines and diagnostics used, the accurate prescription, dispensing, administration and usage of these medicines, and the appropriateness of the counselling and instructions patients receive in order to guide treatment at home.

To take all these factors into account, we expand Obrist and colleagues’ framework [12] by adding a sixth component linked to these “aspects of quality.” This not only allows us to discuss issues of access to medical drugs, but also the degree to which the accessed medicines are used in a way that ensures maximum likelihood of positive health outcomes. We define the 6 dimensions of access to quality healthcare in Table 1 and highlight aspects that influence the agency and choice of individuals to seek and benefit from health provision.

**Table 1:** Definitions of the 5 dimensions of access to quality healthcare (availability, accessibility, affordability, adequacy, and acceptability) adapted from Obrist and colleagues (2007) [12] but expanded to include a sixth dimension that we define “aspects of quality” (in bold) to account for aspects of quality and accuracy of treatment and care.

Dimension	Definition	Factors influencing access to healthcare
Availability	The physical existence of health facilities for patients to attend or of drugs/diagnostics for clients to buy/use.	- What service providers are available? - What drugs/diagnostics are available? - What kind of infrastructure is available and is this sufficient? - Is there an adequate number of trained staff?
Accessibility	The ability of patients to reach, attend, and use the available health service.	- Can patients reach the available facilities? - Is transport available? - Is the service in the patient’s language? - Is the infrastructure of the facility usable? - Are patients seen promptly by a physician when they are at a facility?
Affordability	The ability of patients to pay for health services, including drugs.	- Are prices of drugs/services affordable for patients? - Are transport costs affordable? - Can patients afford the time off work/childcare?
Adequacy	The ability of the organisational structures and processes of the provider to meet patients’ requirements.	- Is the service tailored to patients’ daily schedules and duties? - Is the service in the patient’s language? - Is the facility and its infrastructure well-kept and usable? - Are patients seen by physician in a timely way?
Acceptability	The perceived appropriateness of the form of service provision by the patient.	- Do patients trust service providers? - Do patients feel welcomed and cared for? - Are patients’ expectations met? - Are local understandings and perceptions around illness, and social values considered by the provider?
**Aspects of quality**	The aspects of quality of healthcare products and their provision that influence the ability of the given treatment to produce a positive health outcome. This includes drug quality, accuracy of drug choice, and the appropriateness of counselling and advice.	Drug quality: - Are drugs/treatments of good quality? Accuracy of drug choice and usage: - Are guidelines related to diagnostics and treatment followed? - Are the prescriptions accurate (type/kind of drug, dosage)? - Are treatments/drugs taken accurately (dosage, route of administration, length of course)? Appropriateness of counselling and advice: - Do patients receive the needed advice on how to use or administer drugs/treatment? - Are diagnoses, prescriptions, and instructions communicated to patients in an understandable manner? - Are counselling, information, and instructions for use accurate?

In this paper, we use our expansion of Obrist and colleagues’ framework [12] to review relevant literature from East Africa (focusing on Tanzania, Kenya, and Uganda) in order to discuss the 6 dimensions of access to quality healthcare, including the provision of essential drugs, for humans and animals. We consider both institutional and retail facilities in the public and private healthcare realms, delivering formal or informal services (see Box 1). We discuss how dimensions of access and quality interact in a way that limits patients’ agency and choice during health seeking, and that shapes their practices including those that might impact on AMR emergence. We highlight where shortcomings in equitable healthcare access and service provision lie and what efforts and investments are required to achieve universal health coverage that produces positive health outcomes without exacerbating AMR.

**Box 1. Type of health provision**

For the purpose of this review, we consider the following human and animal healthcare providers:

- **Institutional:** Institutional healthcare providers who perform consultative services for patients in facilities run by medically trained staff.
- **Retail:** Outlets that primarily sell medical products to clients.
- **Public:** Government-led facilities that serve the general public at no profit.
- **Private:** Owned by either private organisations or individuals, who may pursue their own interests, hence often designed as for-profit.
- **Formal:** Official, state-recognised, licensed services and therefore subject to state regulations and controls under the formal health system.
- **Informal:** Not officially recognised or licensed and therefore operating outside the formal health system.

We recognise that the boundaries between these health provision bodies, especially between formal and informal entities, are often unclear, porous, and blurry. For instance, institutional health providers may include a retail shop at their premises, health professionals may work both in public and private facilities, and informalities may occur at all levels [68]. For example, community health workers or dispensers in accredited drug shops often operate at the border of formal and informal care due to limited opportunities for their formal training and certification [68]. All providers we include herewith play a critical role in health provision for humans and animals in the East African context. We therefore discuss them while recognising that different health policy approaches might include or exclude some of them from the formal health system [68].

## Methods

Articles reviewed in this paper were identified by searching (“demand” OR “sale” OR “use” OR “usage” OR “quality” OR “access” OR “accessibility”) AND (“drug*” OR “medication*” OR “treatment” OR “antibiotic*” OR “antimicrobial*” OR “anti-microbial*” OR “antimalarial*” OR “growth promoter*”) AND (“Africa” OR “East Africa” OR “Eastern Africa” OR “Tanzania” OR “Uganda” OR “Kenya”) AND (“human*” OR “animal*” OR “livestock” OR “cattle” OR “cow*” OR “goat*” OR “sheep*” OR “poultry” OR “chicken*” OR “pig*”).

To reduce the number of papers to review in full, terms were searched for in the title or the abstract. Only the last group of terms was searched for in all fields. Two searches were conducted in PubMed and the University of Glasgow’s (UoG) library interface, respectively. After initial sorting, the UoG and PubMed resulting papers were combined and reviewed manually. Inclusion criteria included article in English and set in East Africa. Papers were excluded if they discussed viral diseases, noninfectious diseases, recreational drugs, or access or quality of life without reference to medical drugs. Additional papers were reviewed as identified through snowball methodology from the initial search results. Finally, for broader context, we included and reviewed relevant literature suggested by peers with related content expertise. Overall, 154 publications (86 from the initial search, 22 from reference lists of identified articles, 8 from suggestions, and 37 from searches related to the broader context) were reviewed in full. However, only selected references are provided in this review.

## Result of the literature search

### Availability

In East Africa, human and animal healthcare facilities are unevenly distributed, which leads to inadequate coverage, especially in rural, hard-to-reach areas [14,15]. Many public human health services are severely understaffed [15], and the level of training of human health professionals is variable [10,15,16]. Underfunding and understaffing are widespread also within public veterinary services [2,17,18]. Staff shortages translate into long waiting times for patients, constraining availability of formal human and animal health services for many people [14,17,19]. Furthermore, an inefficient supply and procurement system leaves public health facilities and formal veterinary staff with stockouts or expiry of essential medicines like antibiotics or antimalarials [20,21]. Similarly, diagnostic tools and supplies frequently face low availability or are underused both in human and animal health. For example, a human health service readiness and availability assessment conducted in Tanzania in 2012 found that only 27% of public hospitals were able to perform tuberculosis microscopy and that 40% of dispensaries had malaria diagnostic capacity [22]. Despite their suitability for low-resource and field settings, limited diagnostic capacity extends to low-cost rapid tests that would enable relatively underskilled staff to diagnose human and animal diseases without access to specialised laboratory equipment [22,23]. In higher-tier human and animal healthcare, shortage of staff who can operate the equipment or perform laboratory techniques restrict access to more sophisticated diagnostic tools [23,24]. Long-distance transport of diagnostic samples to specialised laboratories increases turnaround time and costs for patients [10,23].

Where availability of institutionalised human or animal health services is very poor, most people are forced to turn to the retail sector, i.e., pharmacies, agrovet, and drug shops, which are more abundant [17–19,25–27]. In these facilities, staff with professional medical background are not the norm, and restrictions on sales for prescription-only medicines impact drug availability [19,26–28]. In addition, diagnostic capacity to inform treatment is typically absent [29]. Yet, many unlicensed outlets still stock and sell human and veterinary medicines, including antimalarials and antibiotics, thereby increasing the availability of these essential drugs in areas far away from formal health services [26,30,31]. Private retailers have become a very important secondary source of vital drugs in case of stockouts at public human health facilities [31,32]. Similarly, farmers often buy and administer drugs to their livestock themselves or rely on lay personnel when veterinarians are in short supply [17,18,25].

In summary, access to human and animal health services in East Africa is tied to the structure of the available facilities (public/private and formal/informal; see Box 1) that differ in type of services they provide. The mentioned constraints of formal services can lead to diminished access to professional provision of human and animal healthcare. Retail outlets are more abundant and often the only source of drugs for many.

### Accessibility

Accessibility refers to patients being able to physically reach health facilities. Long distances to human health facilities are major hindrances for health seekers, especially when lack of transportation is an additional obstacle [14]. Poor or nonexistent road infrastructure and public transportation, or seasonal weather conditions can exacerbate accessibility problems [33]. The same is true for animal health services where veterinarians and animal health workers need to travel to remote areas in order to attend farmers [34]. Retail shops tend to be located nearer to patients and clients than institutional health facilities, especially in rural areas, and often become the only source of care [17–19,25,31].

### Affordability

Affordability, i.e., cost of treatment, is often cited as a prohibitive constraint for people seeking healthcare [14,19,31–33,35]. Drug purchases make up a substantial proportion of a household’s budget, especially for poor people [36,37]. For example, Machuki and colleagues (2019) found that treating a child with pneumonia in a public health facility had an average cost of US$4.7, with the price of medicines and opportunity costs (caregiver time and forgone wages) making up the biggest part [38]. Generally, purchasing drugs was found to have an impoverishing effect for people in several LMICs [39]. Issues of costs are particularly problematic in the most under-resourced areas, for example, Tanzania and Uganda, where the proportion of the population in danger of falling below the poverty threshold (US$1.25/day) was 60% and 54%, respectively [39].

Affordability heavily influences the type of care or drug clients choose to pursue. People may choose cheaper, nonrecommended, or low-quality drugs over recommended treatments [19,32,35]. If, as a result of inappropriate treatment, the illness persists or is exacerbated by adverse effects, costs escalate even more [36]. To counter affordability issues, certain essential treatments, like antimalarials for children, are available for free in some public health facilities [32,40–42]. However, user fees and diagnostic testing can still pose challenges, especially for the rural poor [10,38]. Transport and waiting times may be too costly or lengthy, during which time the person is unable to work, rendering the service unaffordable for patients [10]. All these expenditures are often paid out of pocket because insurance schemes are rarely functional [43].

Affordability impacted decisions on antimicrobial use in studies in northern Tanzania [34] and Kenya [28]. In Caudell and colleagues’ (2020) study, a Kenyan farmer explained that most money in his household is spent on food and medicines, which leaves little for biosecurity and infection prevention [18]. The same study also mentions costs as a barrier to accessing veterinary professionals [18].

### Adequacy

Adequacy refers to the organisation of health services and how this impacts patients’ access to drugs. For example, restricted opening hours and long waiting times caused by understaffing at institutional facilities can decrease access to formal human and animal healthcare [14,17,19,31,32]. Nonfunctionality of diagnostic equipment or the inability to repair or maintain it can make specific services inaccessible for patients [10,14,23]. In contrast, retail shops selling human and animal health products tend to have longer and more flexible opening hours, including weekends, and have faster service, making them a more adequate source of care compared to other health providers [19,28,31,32].

### Acceptability

Patient expectations and attitudes play a major role in their considerations of what makes a provider or a treatment acceptable to them or not. These often determine which of various treatment or provider options are taken. When patient expectations are not met, this can deter patients from seeking further care in a given facility. Patient dissatisfaction with formal healthcare often results from unreliable drug supply and frequent stockouts [21,35]. Inconsistent treatment within and across facilities, for example, due to different work environments or varying levels of staff training, is a further issue that can potentially decrease acceptance [14,40].

Patients who seek care in the retail sector have more freedom over which treatment they choose and often base their decisions on acceptability rather than appropriateness. Customer preferences and demand influence what drugs are stocked in drug shops. Specific brands or forms of drugs are sometimes preferred, like medicines from western countries or more expensive drugs, because they are considered more effective [10,19]. Patient preference and pressure can also lead physicians in institutional facilities into prescribing specific (antimicrobial) drugs patients are familiar with or believe they require, even without indication [26,44]. Refusal to do so can lower patient trust, satisfaction, and acceptability of the treatment [26,44]. When physicians give in to patient demand, acceptability defines which or how many drugs are dispensed at institutional health facilities [26,45]. In other instances, diagnostic testing to inform treatment is important for patient’s satisfaction [19,46,47].

While most studies address issues of acceptability in the context of human health, farmers’ expectations and preferences are likely to be important also in the treatment of animals. For example, Higham and colleagues’ (2016) study showed that in agrovet stores in the Kenyan Rift Valley, customer choice was considered one of the leading motivations of drug selection [28].

### Aspects of quality

The quality of medicines prescribed or bought as well as the appropriateness and accuracy of drug choice, prescription, dispensing, and use (dosage, route of administration, and course length) all influence if a given drug possesses the ability to effectively treat and cure the patient. More broadly, the quality of treatment provision, i.e., professional counselling and advice by the provider, is an important part of quality of care. In the following section, we expand upon these 3 components.

#### Drug quality

The quality of medicines is an essential aspect of drug-based treatments and their ability to address a given medical condition effectively. Drug safety, efficacy, and good quality are central to achieve the best health outcomes possible. Entirely or partially ineffective drugs do not completely clear pathogens or do not relief symptoms of disease. Therefore, they lead to increased mortality, morbidity, and suffering for patients and animals, as well as higher costs [36,37].

Drugs of poor quality (defined in Table 2) are sold globally and include a wide range of medical products [36,43]. Aggregated observed failure rates of substandard and falsified medicines are increasing worldwide and reported by WHO to be around 10.5% in LMICs [37]. A literature review by Almuzaini (2013) found a median global prevalence of 28.5% of substandard and counterfeit drugs (range 11.0% to 48.0%) with higher prevalence in sub-Saharan Africa and southern Asia [48]. A systematic review by Ozawa and colleagues (2018) found a global prevalence of substandard/falsified products of 19.1% (15.0% to 23.3%) for antimalarials and 12.4% (7.1% to 17.7%) for antibiotics [49].

**Table 2:** Definitions of poor-quality drugs based on the international classification by WHO [43].

Unregistered/unlicensed	Substandard	Falsified
Products that have not undergone evaluation or approval processes	Authorised products that fail to meet specifications or quality standards	Products that deliberately and/or fraudulently misrepresent their identity, composition, or source

Findings regarding drug quality in East Africa are mixed. For example, Kaale and colleagues (2016) found that 92.6% of the medicines, including antimalarials and antibiotics, sold by Tanzanian licensed retailers met quality standards [50]. In contrast, Atemnkeng and colleagues (2007) found only 50.0% of their samples of registered antimalarials from Nairobi, Kenya, to qualify for content requirements [51]. Similarly, Kaur and colleagues (2008) found 12.2% of their antimalarial samples from 21 districts in Tanzania to be of poor quality [52]. Regarding antibiotics, Mwambete and colleagues (2014) found “variability in the effectiveness of antibiotics available” in the northern border region of Tanzania [53], and in Uganda, Kitutu and the Uganda Medicines Transparency Alliance (2017) reported various antibiotic samples that failed a thin layer chromatography (TLC) test [54]. Poor-quality drugs have been detected also in public health facilities, for example, in Kenya, where Wafula and colleagues (2017) documented drug samples (including antibiotics), which did not pass compendial tests [55]. Overall, poor quality of drugs seems to be a bigger concern in informal retail, but data are lacking to make confident statements. Similarly, while very little is known about the quality of veterinary drugs, single studies anecdotally suggest that poor-quality medicines are sold also in this sector [9,17,25,28].

Substandard quality is partly attributed to inadequate transport and storage conditions or poor manufacturing [9,36,51]. Studies suggest that for most drugs, the shelf life changes when stored in equatorial climates [36], especially as some stores or households may not have access to reliable cooling facilities. Furthermore, in-depth quality testing of drugs requires expensive laboratory equipment and specialised staff, making it very costly, especially in resource-constrained settings. Consequently, in these contexts, post-marketing quality surveillance of antimicrobials is largely lacking [36,43].

#### Accuracy of drug choice and usage

Choice, prescription, and dispensing of drugs appropriate to a given health issue, and correct modalities of use are critical, yet they are often considered suboptimal in East Africa in both human and veterinary medicine, irrespective of the source of treatment [10,56,57]. For example, Mboya and colleagues (2018) state that, in Tanzania, antibiotics are bought without prescription and used in incomplete dosages or to treat nonbacterial illnesses in more than 80% of cases [57]. Similarly, in their animal health study in Kenya, Higham and colleagues (2016) reported that all customers either always (15%) or sometimes (85%) ask for specific drugs and that these are often provided without advice on modality of use [28].

In many cases, guidelines on drug prescription, use, and antimicrobial stewardship are either nonexistent or unavailable to those prescribing treatment, especially in the retail sector, or they are inadequate, incomplete, or change regularly [30,45,47,58]. These changes are not communicated effectively; hence, healthcare providers lag behind on the latest recommendations, policies, and guideline updates [58].

However, even where such treatment guidelines are actively implemented, depending on available resources and work environment, healthcare providers are not always able to follow these procedures. For example, at public human and animal facilities, the choice of drugs should be informed by a diagnosis, but tools for confirmation are often unavailable [16,23,59] or underused [10,41,58]. Therefore, patients are often treated on the basis of symptoms and clinical signs instead [23,28,47,58]. In case of diagnostic uncertainty, healthcare providers tend to play it safe and prescribe antimicrobials. While this approach gives patients access to lifesaving drugs, it could conversely lead to a misalignment between drugs prescribed and the condition the patient or sick animal presents with [10,26,47]. Furthermore, even when diagnostics are available, physicians do not always trust negative test results, particularly if prior self-treatment by the patient is suspected [26,60]. Patient pressure and demand [18,26,28,44,56], patient’s affordability issues [14,19,28,31–33,35], and stockouts [20,21,32] further complicate drug choice in all human and animal health settings. Consequently, these factors may lead to less positive health outcomes, which, in turn, can create mistrust or reluctance in patients and impair acceptability [13,47].

When self-medicating themselves or their animals, farmers and patients may use drugs for the wrong condition [25,57,61], and incomplete courses of treatment and under or overdosing are commonly reported [17,18,35]. Treatment may be stopped prematurely because of symptom improvement [18,26,35], side effects [26], or because patients/farmers want to save medicine for later or share it with others [26,35]. Use of low-dose antibiotics mixed with feed for growth promotion is common in commercial farming [9], while it is less practised in traditional livestock production systems, which are the most predominant in East Africa [17,28].

#### Appropriateness of counselling and advice

Patients and livestock owners do not always receive appropriate advice on drug choice, administration, dosage, frequency, and length of medication course [18,27,28,62]. Obtaining such information in informal outlets can be even harder, as dispensers often do not have formal medical training [27,28]. Studies assessing knowledge, attitudes, and perceptions of antibiotic use and AMR have shown that even trained human and veterinary medical staff do not always have sufficient knowledge or awareness of AMR in Africa [63,64]. This impacts patient safety and appropriateness of treatment. Thus, problems with appropriateness of service provision have several causes. For example, understaffing creates an inadequate patient–provider ratio in formal services, which increases staff workload [16,18]. Resulting limitations in counselling time, along with the stress that providers face, impact the quality of care [45]. Variable levels of training and supervision of human and animal health professionals and shop dispensers further impair ideal health provision, impacting health outcomes [10,27,45,56].

## Discussion

### Increasing access and ensuring optimal use

**Fig 1:**
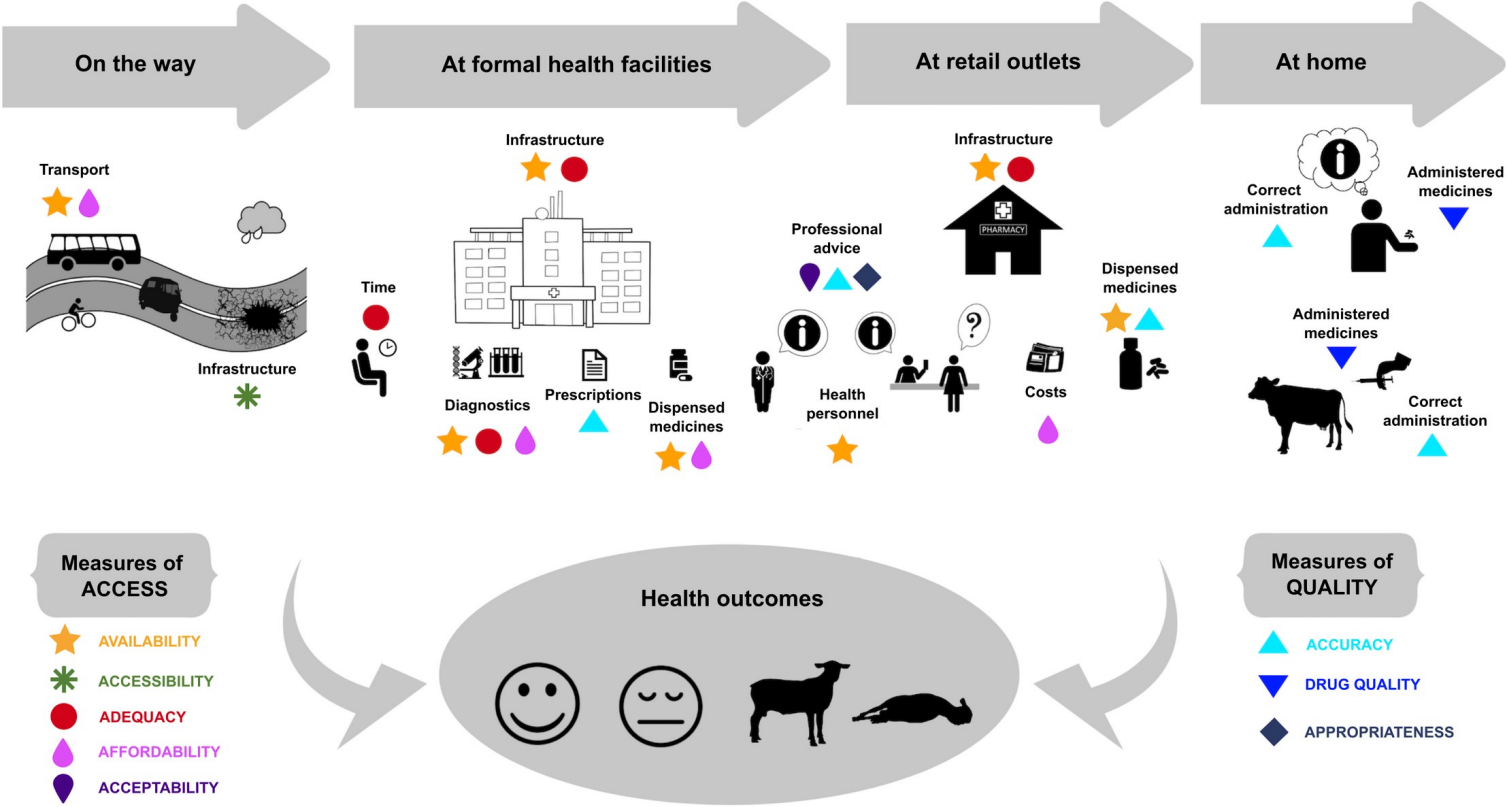
Components of the healthcare system and health-seeking process. The shapes show where shortcomings in access or quality of human and animal healthcare could happen and impair ideal health provision. They also indicate the dimension of the framework affected.

As we discuss above, gaps in equitable access to drugs and the quality of their provision still remain in East Africa. There are many instances in which inadequacies may manifest during the health-seeking process for people and animals (Fig 1*)*. Infrastructural, economic, organisational, and social barriers exist at all levels and involve all 6 dimensions of access and quality of human and animal healthcare.

While access to quality drugs is critical for positive health outcomes, their overuse increases the risk of AMR development. Pharmaceuticalisation of healthcare means that patients perceive the provision of drugs as the most important component of healthcare [26,56]. Commodification of care, patient preference, diagnostic uncertainty, hence poorly informed drug choice—all driven by limited access to and uneven quality of care—lead to polypharmacy, overuse, and overprescription of antimicrobials in the cases when these can be accessed. Additionally, self-administration with suboptimal drug dosage or course length results in patients over or underdosing themselves or their animals. Such practices are a consequence of variable availability and quality of formal, professional healthcare, information, and training of staff. Along with raising levels of substandard and falsified medical products on the market, all of the above contribute to AMR risks in LMICs.

### Recommendations for future action and research

Concerns around AMR should not compromise optimal access to lifesaving drugs. The biggest challenge therefore lies within balancing access to essential medicines and their over and suboptimal use [10]. As Mendelson and colleagues (2016) write, “Limited access to and overuse of antimicrobials often coexist within one health system and cannot be tackled by targeting any one of these challenges in isolation” [10].

Our framework integrates all dimensions of access and quality of human and animal healthcare to highlight existing shortcomings in the system resulting from the interplay of all these complex factors (Fig 1). We advocate that, to balance the need for universal and equitable access and for accurate, nonexcessive usage of drugs, actions should target all 6 dimensions. Interventions involving key players in the system, from patients and farmers to healthcare staff and policy makers, offer opportunities to improve health outcomes while simultaneously counteracting the AMR crisis (see Box 2 for examples).

**Box 2. Recommended actions to improve health outcomes while counteracting AMR**

Assuring quality of medicines

- Reporting cases of substandard, unregistered, or falsified drugs [43].
- International harmonisation of laws and jurisdiction about drug manufacturing, marketing, and distribution [36,43].
- Implementation of drug quality testing and monitoring at all stages in the supply chain [36,43].
- Establishing adequate storing capacity at point of care facilities [36,43].
- Enforcement of state drug quality regulations [36,53].
- Establishing international networks to enable exchange of information and skills [43].

Making medicines affordable

- Free dispensing of essential medicines for vulnerable groups at public health facilities [42].
- Universal affordable insurance schemes [43].
- Subsidy schemes to make essential medicines affordable in the private sector (see The Affordable Medicines Facility–malaria (AMFm) programme for an example) [41].
- Implementation of awareness and social marketing campaigns to nudge patients into using recommended, affordable, and safe services [14,21,32].

Ensuring availability of medicines and appropriate provision at point of care

- Optimisation of the drug supply chain to public services to avoid stockouts [20,36].
- Ensuring availability of diagnostics to enable evidence-based, focused drug use [10,26,40].
- Investments in infrastructure and diagnostic capability, especially in rural areas [12,65].
- Training and employment of additional staff to reduce waiting times and workload, allowing more time for appropriate counselling and advice [15,17].
- Integration of training focusing on AMR in medical (human and animal) schools [63,64].

Continued education, patient management, communication, and public health literacy

- Distribution and clear communication of national treatment guidelines in all sectors [10].
- Formation of adequate training, supervision, and feedback loops for health professionals at all levels [10,26].
- Regular (refresher) courses for drug dispensers to disseminate/reinforce knowledge and messages around drug dispensing, diagnostics, and stewardship [14,26,44].
- Inclusion of communication skills in the training of drug dispensers to help them cope with patient demand [26].
- Awareness campaigns and health education for the public emphasising the need for professional advice on human and animal health matters and where to find it. Awareness creation for patients and farmers of what good healthcare is and what they can/cannot expect from different healthcare providers [21,26].

Inclusion of informal care into health system planning

- Officially recognise retail and community-based providers as an essential part of the public health sector and integrate them into formal health planning [31,66].

## Conclusions

Well-equipped and trained health providers remain the backbone of effective healthcare. Understanding the constraints health seekers face in a complex health system comprising multiple actors will help tailor interventions to relevant issues. Efforts to increase access to healthcare need to be combined with appropriate dispensing and accessible diagnostic tests to enable accurate diagnosis and targeted usage of drugs. Good governance is also needed for local, national, and international policy development around drug quality and use. Further research elucidating the link between human and veterinary antimicrobial use and AMR will inform guidelines for drug prescription, dispensing, and use. Stewardship programmes and surveillance systems need to be designed, evaluated, and updated regularly on the basis of this information [10,59]. Incorporating training in these processes in medical, nursing, and pharmacy schools, but also tailored to informal providers, will increase health provider’s knowledge about antimicrobials and how best to deliver them to mitigate AMR risks [14,44,63,64]. More broadly, essential preventive measures enabled by water, sanitation, and hygiene infrastructure and quality as well as deployment of mass vaccination and biosecurity measures on farms would reduce the need and demand for antimicrobials at source [10,59]. The focus should lie on scaling up successful programmes (for examples, see [14,21,41,46,65,66]), making quality healthcare a priority and finding sustainable ways of funding. East African countries could benefit greatly from international collaborations and knowledge and skill transfer. For instance, much could be learnt on how to ensure broad and equitable access to drugs through public health infrastructure while minimising informal provision from South Africa’s experiences of tackling human immunodeficiency virus/acquired immunodeficiency syndrome (HIV/AIDS). Lessons from implementing AMR surveillance in this part of Africa would also be of value [67].

Ultimately, broad access to good quality human and animal healthcare and targeted use of drugs will reduce the burden of illness, particularly among disadvantaged and vulnerable groups, while simultaneously decreasing risks of AMR emergence.

### Limitations

This study is not a systematic review, and we have not undertaken methodical classification of evidence. Because of the sheer number of studies about access to, demand for, and use and quality of antimicrobials, and about AMR, it was necessary to limit certain search terms to title or abstract. However, in the process of reviewing the identified papers, we reached a point of saturation with no further issues emerging around antimicrobials and AMR than those described in this paper. Therefore, we are confident that the selected papers and topics presented here are a good representation of the situation of access to and quality of antimicrobial drugs in human and animal healthcare in East Africa. However, one aspect our search clearly identified relates to a general overrepresentation of research on human health (135/154 studies) over animal health (17/154 studies) with only 2 out of 154 studies integrating animal and human health processes. In addition, within the 135 human health studies, research on antimalarials (91/135 studies) predominated compared to antibiotics (66/135 studies) and other kinds of drugs (29/135 studies). This highlights the need for further research examining antimicrobial access and quality in animal health more specifically, and for a greater focus on antibiotics.

Finally, our results are geographically confined to Tanzania, Kenya, and Uganda and may not apply to other countries or regions. Nevertheless, our framework can potentially be applied to evaluate healthcare-related shortcomings of access and quality in any LMIC context.

**Key Learning Points**

- We use a framework containing 6 dimensions of access and quality of healthcare, including availability, accessibility, affordability, adequacy, acceptability, and aspects of quality, to explore literature from East Africa.
- We find shortcomings in access to basic healthcare and quality of care provision, which span all 6 dimensions of the framework.
- Such inadequacies lead to suboptimal health outcomes and can potentially fuel problematic practices around antimicrobial use, hence antimicrobial resistance (AMR).
- In order to balance the need for better access to essential medicines and the threat of AMR, interventions should target all 6 dimensions of access to quality healthcare.
- In particular, the dimension aspects of quality, including the quality of medicines and provision of health services, is an important aspect to consider in health policy and interventions targeting the AMR crisis.

**Top Five Papers**

1. Obrist B, Iteba N, Lengeler C, et al. Access to healthcare in contexts of livelihood insecurity: A framework for analysis and action. PLoS Med. 2007;4:1584–8. doi: 10.1371/journal.pmed.0040308
2. Mendelson M, Røttingen JA, Gopinathan U, et al. Maximising access to achieve appropriate human antimicrobial use in low-income and middle-income countries. Lancet. 2016;387:188–98. doi: 10.1016/S0140-6736(15)00547-4
3. Almuzaini T, Choonara I, Sammons H. Substandard and counterfeit medicines: A systematic review of the literature. BMJ Open. 2013;3. doi: 10.1136/bmjopen-2013-002923
4. Hetzel MW, Iteba N, Makemba A, et al. Understanding and improving access to prompt and effective malaria treatment and care in rural Tanzania: The ACCESS Programme. Malar J. 2007;6:1–15. doi: 10.1186/1475-2875-6-83
5. Dillip A, Kimatta S, Embrey M, et al. Can formalizing links among community health workers, accredited drug dispensing outlet dispensers, and health facility staff increase their collaboration to improve prompt access to maternal and child care? A qualitative study in Tanzania. BMC Health Serv Res. 2017;17. doi: 10.1186/s12913-017-2382-1
